# Supplementary material for: Whole-organism spatial transcriptomics at single-cell resolution in C. elegans
Source: bioRxiv. 2026 Apr 11:2026.04.09.717568. Preprint. [Version 1] doi: 10.64898/2026.04.09.717568 (PMC13081990; doi:10.64898/2026.04.09.717568)
Supplement: Supplement 1 [file NIHPP2026.04.09.717568v1-supplement-1.pdf]

bioRxiv preprint doi: https://doi.org/10.64898/2026.04.09.717568; this version posted April 11, 2026. The copyright holder for this preprint (which was not certified by peer review) is the author/funder, who has granted bioRxiv a license to display the preprint in perpetuity. It is made available under aCC-BY 4.0 International license.

Supplementary figures

DATABASE	Gene set used	Number of genes	Article title
Ebbing A. et al., (2018)	Male tail enriched	123	Spatial Transcriptomics of *C. elegans* Males and Hermaphrodites Identifies Sex-Specific Differences in Gene Expression Patterns
Kaletsky R. et al., (2018)	Neuron enriched	867	Transcriptome analysis of adult *Caenorhabditis elegans* cells reveals tissue specific gene and isoform expression
Spencer W.C. et al., (2011)	Neuron enriched	547	A spatial and temporal map of *C. elegans* gene expression
Kim B. et al., (2016)	Mod 1,2,3	7101	Gene function prediction based on developmental transcriptomes of the two sexes in *C. elegans*
Kim B. et al., (2016)	Mod 1, 2	3628	Gene function prediction based on developmental transcriptomes of the two sexes in *C. elegans*
Kim B. et al., (2016)	Male enriched	1751	Gene function prediction based on developmental transcriptomes of the two sexes in *C. elegans*
Kim B. et al., (2016)	Mod 1,2,3 GO analysis	123	Gene function prediction based on developmental transcriptomes of the two sexes in *C. elegans*

Supplementary Table 1. Summary of public RNA-seq datasets analyzed for sequential smFISH target gene selection. RNA-seq datasets from publicly available sources were examined to identify candidate differentially expressed genes between male and hermaphrodite *C. elegans*. Kim B. et al., (2016) dataset was partitioned into 27 co-expression modules (Mod), each representing a distinct gene expression program, from which Mods were utilized. The resulting candidate gene list was used to design the sequential smFISH probe pool.

bioRxiv preprint doi: https://doi.org/10.64898/2026.04.09.717568; this version posted April 11, 2026. The copyright holder for this preprint (which was not certified by peer review) is the author/funder, who has granted bioRxiv a license to display the preprint in perpetuity. It is made available under aCC-BY 4.0 International license.

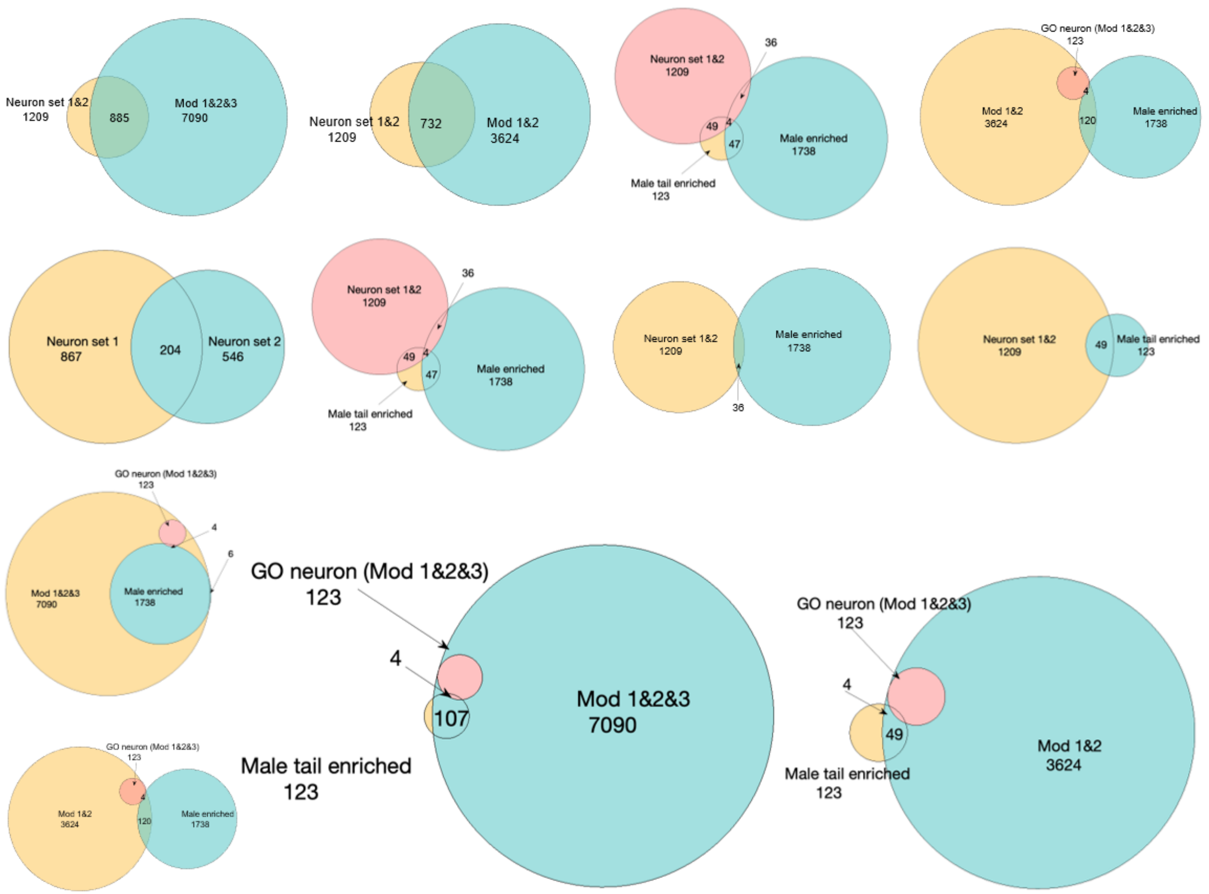

Supplementary Figure 1. Summary of public RNA-seq datasets analyzed for sequential smFISH target gene selection. RNA-seq datasets from publicly available sources were examined to identify candidate differentially expressed genes between male and hermaphrodite *C. elegans*. Kim B. et al., (2016) dataset was partitioned into 27 co-expression modules (Mod), each representing a distinct gene expression program, from which Mods were utilized.
